# DEMENTIA TRAJECTORIES, MEDICAID COVERAGE, AND HEALTHCARE SERVICES USE

**DOI:** 10.1093/geroni/igac059.526

**Published:** 2022-12-20

**Authors:** Wassim Tarraf, Marc Garcia

**Affiliations:** Wayne State University, Detroit, Michigan, United States; Syracuse University, Syracuse, New York, United States

## Abstract

The evidence base linking dementia risk trajectories to transitions in Medicaid coverage and levels of health services is underdeveloped. We use Health and Retirement Study (2007/08-2015/16) data on adults 70-years and older (Unweighted N=8,227) at baseline to test how longitudinal dementia classifications (Langa-Weir), over 8-years, influence change in Medicaid coverage, nursing-home use (NHU) and inpatient hospitalizations (IH). We fit Joint Growth and Discrete-time Survival Mixture models to generate longitudinal risk classifications for dementia accounting for survival, and generalized linear models to test associations of these classifications with change in Medicaid coverage, NHU, and IH. Average baseline age was 78.4 years (SD=7.1), 3-in-5 were female, 1-in-4 had less than high school education, and 4-in-5 were non-Hispanic Whites. A three-class solution (C1=high dementia prevalence and mortality risk (8.6%), C2=low prevalence (68.3%), and C3=accelerated dementia prevalence and mortality risk (23.1%)) provided the best fit to the data. We observed substantial increase in rates of for Medicaid coverage for C1 (21%; 95%CI=[18-25] to 51%[36-65] among survivors) and C3 (12%; 95%CI=[10-14] to 32%[27-37]), but not C2 (6% to 8%). NHU also accelerated substantially from 10 to 37% in C1 and 7 to 38% in C2. Rates of inpatient hospitalizations remained stable over time for all groups, with C1 and C2 being more likely to be hospitalized and have multiple re-admissions. Estimates were differentially attenuated through adjustment to covariables. We report important longitudinal dementia risk classifications, profile their socioeconomic and health attributes, and identify differential associations with critical health policy outcomes (Medicaid coverage and healthcare utilization).

